# DeepSurvNet: deep survival convolutional network for brain cancer survival rate classification based on histopathological images

**DOI:** 10.1007/s11517-020-02147-3

**Published:** 2020-03-02

**Authors:** Amin Zadeh Shirazi, Eric Fornaciari, Narjes Sadat Bagherian, Lisa M. Ebert, Barbara Koszyca, Guillermo A. Gomez

**Affiliations:** 1 Centre for Cancer Biology, SA Pathology and University of South Australia, UniSA CRI Building, North Terrace, Adelaide, SA 5001 Australia; 2 Department of Mathematics of Computation, University of California, Los Angeles (UCLA), Los Angeles, CA USA; 3 Department of Ophthalmology, Mashhad University of Medical Sciences, Mashhad, Iran; 4 SA Pathology, Royal Adelaide Hospital, Adelaide, Australia

**Keywords:** Deep learning, Survival rate, Brain cancer, Histopathological images, Classification, Convolutional neural networks

## Abstract

Histopathological whole slide images of haematoxylin and eosin (H&E)-stained biopsies contain valuable information with relation to cancer disease and its clinical outcomes. Still, there are no highly accurate automated methods to correlate histolopathological images with brain cancer patients’ survival, which can help in scheduling patients therapeutic treatment and allocate time for preclinical studies to guide personalized treatments. We now propose a new classifier, namely, DeepSurvNet powered by deep convolutional neural networks, to accurately classify in 4 classes brain cancer patients’ survival rate based on histopathological images (class I, 0–6 months; class II, 6–12 months; class III, 12–24 months; and class IV, >24 months survival after diagnosis). After training and testing of DeepSurvNet model on a public brain cancer dataset, The Cancer Genome Atlas, we have generalized it using independent testing on unseen samples. Using DeepSurvNet, we obtained precisions of 0.99 and 0.8 in the testing phases on the mentioned datasets, respectively, which shows DeepSurvNet is a reliable classifier for brain cancer patients’ survival rate classification based on histopathological images. Finally, analysis of the frequency of mutations revealed differences in terms of frequency and type of genes associated to each class, supporting the idea of a different genetic fingerprint associated to patient survival. We conclude that DeepSurvNet constitutes a new artificial intelligence tool to assess the survival rate in brain cancer.

**Electronic supplementary material:**

The online version of this article (10.1007/s11517-020-02147-3) contains supplementary material, which is available to authorized users.

**Graphical abstract:**
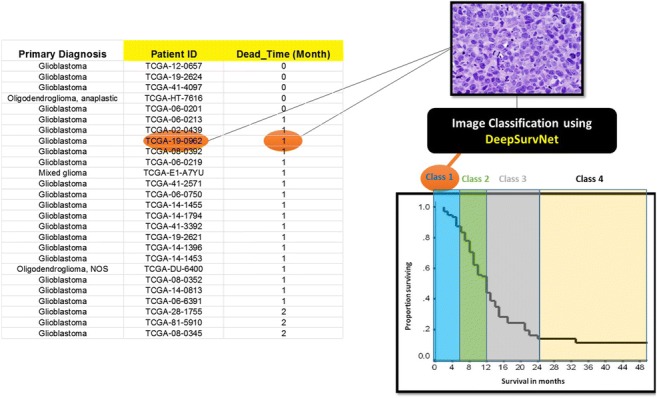
A DCNN model was generated to accurately predict survival rates of brain cancer patients (classified in 4 different classes) accurately. After training the model using images from H&E stained tissue biopsies from The Cancer Genome Atlas database (TCGA, left), the model can predict for each patient, based on a histological image (top right), its survival class accurately (bottom right).

## Introduction

Brain cancer patient classification is mainly based on histopathological images that can accurately identify the type of cancer as well as genetic tests [1, 2]. However, recent single cell RNA seq experiments performed in GBM biopsies [3–8] have challenged these models, pointing out that the reliability of these methods and its use in personalized medicine strongly depends on how much we know on these different type of cancers (i.e. cancer cell subtypes within a tumour) and how many therapies for their individual treatment we have available and whether these target all or none of such cancer cell populations [9]. Thus and as we certainly are still progressing on the molecular determinants that contribute to the aggressiveness of glioblastoma, the current brain cancer classification methods (either based on histological and/or genetic approaches) so far have shown not being sufficient to provided a complete picture on how this can be used to predict (i) survival, (ii) response to treatment and (iii) the development of more personalized treatments [10], which is clearly evident by the following: (i) the lack of development of new treatments for brain cancer patients, in particular, those patients affected by grade IV glioma [10]; (ii) the lack of improvement in brain cancer treatments and patients outcomes (i.e. survival) in the last 30 years [11]; and (iii) the lack of personalized treatments in the clinic, where most oncologists subject patients to the Stupp protocol and knowledge-based on IDH gene mutations and MGMT methylation [12].

Thus, we feel that in addition to the classification of brain tumours that have been done so far, it is also equally important to stratify brain cancer patients based on their survival characteristics and which will permit us to clinicians to tailor both the timing and the type of treatments to patients [12, 13]. This will, for example, be helpful or avoid overtreating those patients with more stable disease. Moreover, classification of brain tumours as a function of brain cancer survival will help us to reveal key characteristics that make these tumours very aggressive and for those patients that present long survival, what are the molecular signatures that contribute to it [13].

Thus, survival rate analysis has become essential for clinicians to select the best treatment methods based on the patient’s clinical data [14, 15]; and survival predictor models have been developed in oncology to investigate the relationship between information obtained at the time of diagnosis and the overall patient’s survival [16]. This has been further facilitated by the recent access to large datasets of digital images, e.g. The Cancer Genome Atlas (TCGA), at the moment of diagnosis, including those from computed tomography (CT), magnetic resonance imaging (MRI) and whole slide pathological imaging (WSI), which have allowed researchers to investigate patient’s survival based on the information contained in these images [17–20]. For example, Tomczak et al. [21] collected > 2000 lung cancer WSIs, and others established a relationship between the information stored in the pathological images and survival rates [22, 23].

Thus, a different group of models for prediction of the patient’s survival based on the histopathological information collected at the moment of diagnosis have emerged. One group correspond to accurate prediction of the patient’s survival that is related to the traditional hazard models and which are based on the Cox model [24] and its derivations [25, 26]. These consider a linear combination of covariates to predict the risk of the patient’s death with nonlinear functions related to the risk [27]. Another group is based on artificial intelligence and deep learning, on which deep convolutional neural networks (DCNN) are used for the analysis of biomedical imaging and applied to recognition, classification and prediction tasks [28–31]. Numerous examples that use DCNNs have been reported recently to predict the survival rate based on pathological images including Katzman et al. [32] who put forwards for the first time deep fully connected network, namely, DeepSurv, to predict survival rate based on structured clinical data (non-images data) and Zhu et al. [27] who used a modified DCNN, namely, DeepConvSurv, on the unstructured data (867 lung cancer WSIs pathological images) to predict the survival rate. In particular, they changed the DCNN loss function in their model to negative partial log likelihood, and as a result, the output of their network measured the risk value for each patient. In their work, they reported a concordance index (c-index) of 0.63 as their model evaluator. Zhu et al. [33] applied a WSI-based model (viz. WSISA) to predict survival state in lung cancer as well as in glioblastoma (c-index 0.7, 0.64 for lung cancer and glioblastoma, respectively), although in a limited manner as (i) WSIs from TCGA with 0.5-μm/pixel (p) resolution were downloaded, and patches of 512 pixels × 512 pixels (512 × 512) size were extracted haphazardly, implying that 54% of the publicly available data was outliers in their analysis, and (ii) high-level semantic information could not be detected in their model. Tang et al. [34] also used DCNN-based model (viz. CapSurv) to predict survival rate in lung and a specific type of brain cancer (glioblastoma) considering patches of 256 × 256 extracted from WSIs from TCGA and applied a new loss function, namely, survival loss, to improve the accuracy (c-index 0.67) of the predictive model.

In addition to accurate prediction of the patient’s survival, supervised machine learning–based algorithms are also used for *classification* [35, 36] where input values (e.g. an image associated to clinical record) are assigned to an output class (e.g. survival within a given time period after diagnosis). Classifiers offer the possibility of predicting with high accuracy the class to which a group of patients belong (e.g. time period after diagnosis) compared to accurate prediction of the patient’s survival methods that are less precise and works inefficiently. As a novel example, Kolachalama et al. [37] utilized DCNN to classify the survival rate of three types of kidney cancer based on WSIs. In their model, the inputs were WSIs without any extracted patches, a computationally very demanding task, and the outputs were three classes of survival rates including 1 year, 3 years and 5 years whose results (area under curve as a classifier evaluator metric) achieve 0.878, 0.875 and 0.904, respectively.

In this work, we use DCNN for classification of brain cancer survival using whole slide histopathological images obtained from haematoxylin and eosin(H&E )-stained biopsy tissue sections, since no models were reported previously for classification of survival rates of brain cancer patients (see [38] for a comprehensive review on brain cancer classification using deep learning methods and MRI imaging). Moreover and although research is progressing on the molecular determinants that contribute to the development and growth of brain tumours, including glioblastoma, the most aggressive form, current classification approaches (either based on histological and/or genetic tests) do not directly focus on the survival of patients [1, 2, 10] and have not yet provided a complete picture on how “brain cancer type classification” can be used to predict (i) survival and (ii) response to treatment and (iii) help the development of more personalized treatments.” In order to address this problem of brain cancer classification based on survival, we put forwards deep survival convolutional network (DeepSurvNet) as a novel classifier approach based on DCNN. Like the other models, we used patches derived from WSIs as inputs in our model, and we trained and tested our model based on WSI images available from TCGA. In addition, we were able to generalize the results of our model by further applying it to a completely independent dataset of H&E images derived from tumour biopsies collected locally by SA Pathology, the South Australian state pathology service. Thus, DeepSurvNet allowed us for the first time to (1) accurately (> 99%) classify brain cancer survival rate directly from the WSIs and (2) validate our TCGA-trained model in an independent and local cohort of patients. The experimental results illustrate that DeepSurvNet model is a distinguished classifier and open a new horizon in the field of survival analysis.

## Methods

### Construction, training and testing of DeepSurvNet

**Fig. 1:**
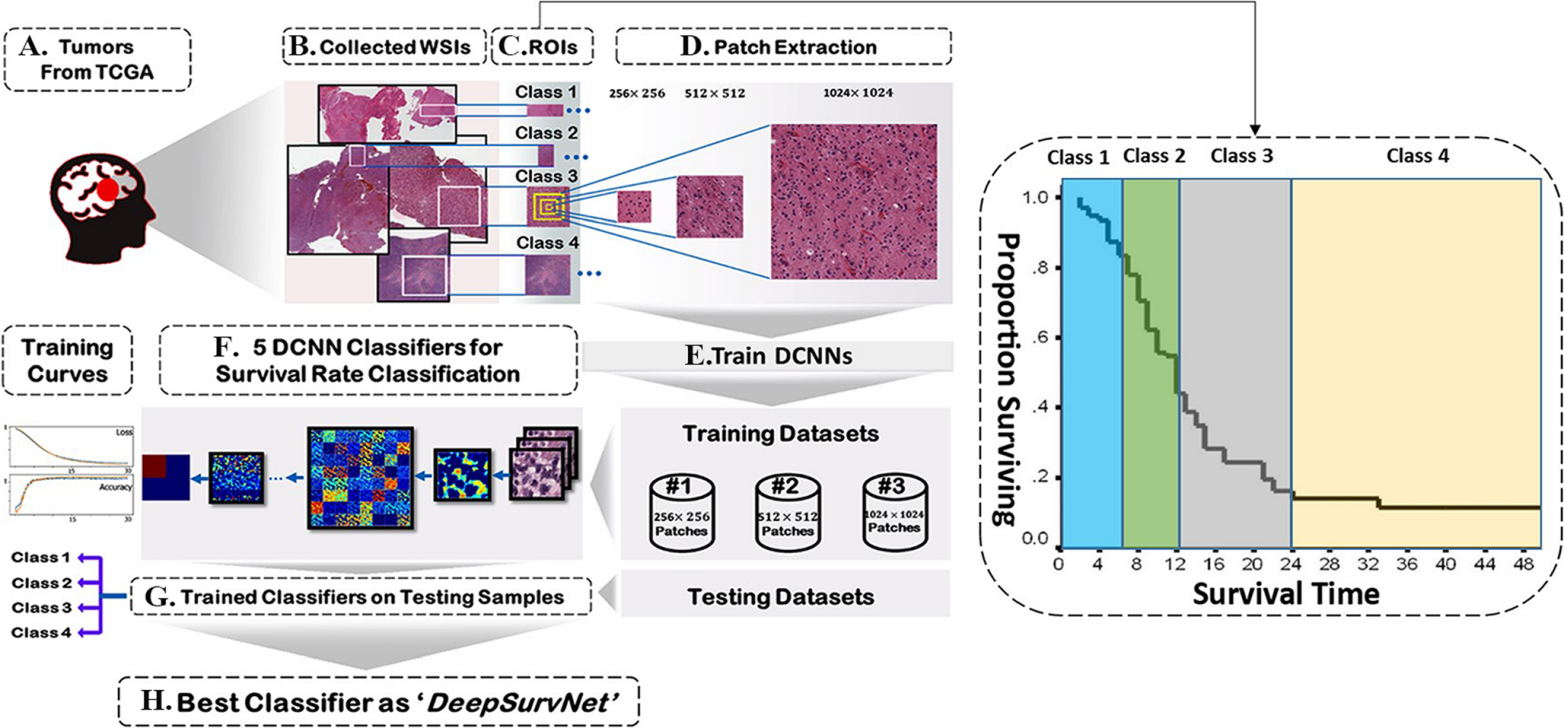
Workflow for construction, training and testing of DeepSurvNet using H&E-stained histopathological images of brain tumours available at TCGA (https://portal.gdc.cancer.gov/projects). The accuracy of all the classifier models is dependent on the image preprocessing steps *b* to *d* on this

Figure 1 presents the steps (*a* to *h*) involved in the construction, training and testing of DeepSurvNet, which are described below.

#### Datasets used for training, testing and validation of deep learning classifiers (Fig. 1a)

We considered two different datasets for the classification of survival rates in patients who suffered from different types of brain cancer including glioblastoma multiform, mixed glioma, oligodendroglioma and astrocytoma. The first dataset is derived from 490 brain cancer patients and is publicly available from TCGA [39] and was used to train and test all the classifier models of survival rates. It is important to mention that within this dataset, slides – and therefore WSI – for each patient contain several tissue sections of the same biopsy, and all of these were used to train and test the classifiers. The second dataset was derived from 9 glioblastoma patients who underwent surgical tumour resection within the South Australian public hospital system. Tumour biopsy specimens were accessed from archival material stored at SA Pathology (the state pathology service), and survival time was calculated based on electronic medical records. Formalin-fixed paraffin-embedded biopsy tissues were sectioned and stained with H&E according to standard protocols at SA Pathology and imaged at 0.5-μm/pixel resolution using a Zeiss AxioImager.M2 microscope equipped with an EC Plan-Neofluar 40x/0.75 M27 Objective and an AxioCam Mrc camera. We used this dataset for an independent test and to monitor the efficiency of our model (i.e. this data was not used for training of the model, for which only TCGA datasets were used).

#### Patients’ database creation: removing outliers and extraction of tumour regions of interest (ROIs) from WSIs (Fig. 1b)

937 WSIs from 490 brain cancer patients were downloaded from TCGA. These were visually explored, and those WSIs that are useless for further analysis because they are corrupted, present marker annotations that cannot be removed, are of low-resolution or lack of clinical information (time of decease after diagnosis) were removed, which left 654 WSI from 445 cases available for further analysis. Guided by the pathologist, we further inspect the data for optimum extraction of several tumour ROIs from each WSI. The total number of extracted ROIs was 849 from the 445 cases. We used this result to create a curated database containing all the patients’ clinical output information including the patients’ ID, mutated genes, and time between brain cancer diagnosis and disease. This database is directly related to all the extracted ROIs used in our work and is available from the authors upon request.

#### Definition of different classes for survival (Fig. 1c)

For classification, we have considered 4 classes. These classes are related to the patients’ history of their time between brain cancer diagnosis to death which was extracted from patients clinical history available from TCGA. Thus, in classes I, II, III and IV, there are respectively 217 ROIs (related to patients with survival time after diagnosis between 0 and 6 months), 210 ROIs (related to patients with survival time after diagnosis between 6 and12 months), 277 ROIs (related to patients with survival time after diagnosis between 12 and 24 months) and 145 ROIs (related to patients with survival time after diagnosis greater than 24 months). Thus, the number of classes and ROIs in each one is sufficiently large for training the DCNN classifiers which are known to be extremely data hungry throughout the training phase [40].

#### Patch extraction from ROIs and patch standardization (Fig. 1d)

ROIs allocated to each class are large in size, and processing them directly is computationally demanding. Thus, for training and testing purposes, we have extracted ROI subregions or “patches” of different sizes 256 × 256 (218,760 patches), 512 × 512 (38,963 patches) and 1024 × 1024 (8657 patches) and compared them to know which can detect more features from the ROIs. For supervised machine learning tasks (e.g. classification), each patch is allocated to a class with a specific label, which results in 4 labels as outputs, and each label is related to each class. Table 1 shows a summary of the number of extracted patches with different sizes for each class.

**Table 1:** Patch extraction from ROIs for each class

	No. of patients	No. of ROIs	No. of patches (256 × 256)	No. of patches (512 × 512)	No. of patches (1024 × 1024)
Class I	95	217	49,705	8778	1921
Class II	96	210	53,428	9480	2099
Class III	133	277	74,326	13,287	3004
Class IV	121	145	41,301	7418	1633
Total	*445*	*849*	*218,760*	*38,963*	*8657*

Finally, as TCGA derived images present variable levels of colour intensities, we standardize their intensities by applying the following formula to each pixel:

1 $$ {P}^{\hbox{'}}=\frac{P-\mu }{\sigma } $$

where *P*^*′*^ and *P* are standardized and original patches, respectively. Also, *μ* and *σ* are the average and standard deviation of all values in the original image patch.

#### Training, validating and testing datasets and DCNN-based classifiers (Fig. 1e, f)

**Fig. 2:**
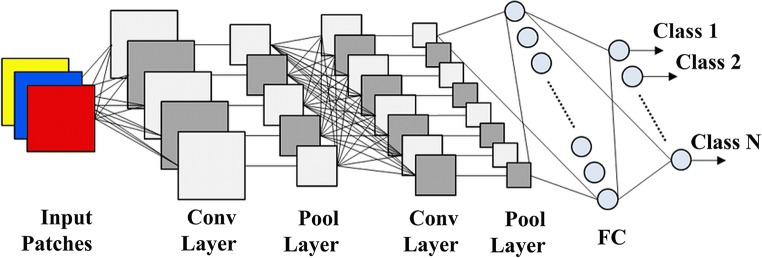
Simple CNN structure with fully connected layers

For each specific patch size extracted from TCGA dataset, we have divided all the patches into three different cohorts including training (80%), validating (18%) and testing (2%). An example of an early CNN structure can be seen in Fig. 2. The early basic architectures popularized by AlexNet [41] loosely follow a pattern of alternating between convolutional layers (Conv Layer) and pooling layers (Pool Layer). The intention is to “learn” features from input layers via convolutional layers and reduce the spatial complexity via pooling layers. Subsequent iterations of these operations distil a set of features that are enrolled into a fully connected (FC) layer which are computed to output classes.

In more modern architectures such as MobileNetV2 [42], FC layers are largely outdated in favour of 1 × 1 convolutions. More performant patterns have also been developed such as residual layers which utilize skip connections introduced in ResNet50 [43].

#### Five DCNN-based classifiers for brain cancer survival rate classification (Fig. 1g)

In order to classify different classes of survival rates based on different sizes of patches, we have considered the most popular DCNN classifiers in image recognition task including VGG19 [44], GoogleNet [45], ResNet50 [43], InceptionV3 [46] and MobileNetV2 [42]. We compared all the results derived from each of these models, and the best-performing model was then used as the engine for DeepSurvNet.

##### VGG19

In 2014, Visual Geometry Group (VGG) in the Oxford University presented A DCNN classifier model named VGG [44] in the ILSVRC [47] challenge and won the image classification tasks using the VGG model. There are several architectures of VGG with different layers, two of which are very popular. The first one is a 16-layer (VGG16), and the other is a 19-layer (VGG19). We use VGG19 as a classifier for survival rate classification task in this study.

##### GoogleNet

In 2014, Szegedy et al. [45] from Google introduced a new conception, namely, Inception, in their article and called their model GoogleNet. In this 22-layer deep network, they have applied filters with different sizes 1 × 1, 3 × 3 and 5 × 5 in the Inception modules. The aim of using such multiple convolutions in the Inception modules would be to feature extraction in different levels. After stacking the outputs of these filters along the channels, they are ready for further layers.

##### ResNet50

In 2015, He et al. from Microsoft introduced the ResNet architecture and demonstrated that using the residual modules, we can train very deep convolutional networks with standard stochastic gradient descent (SGD) method [43]. Among all different kinds of ResNet models, the ResNet50 is very popular since it has simpler structure than the other forms, a reason why we use it in this study.

##### InceptionV3

As mentioned earlier, GoogleNet introduced the Inception architecture or Inception V1. Afterwards, Inception module was purified in various ways and other architectures are introduced by Google as Inception vN where N is the Inception version. The Inception V3 [46] architecture adds new features to the inception module to increase the accuracy of the ILSVRC classification task.

##### MobileNetV2

Another successful approach of DCNN-based classifiers is MobileNetV2 [42] introduced by Sandler et al. from Google in 2018. Although MobileNetV2 is a new idea elicited from MobileNetV1 [48], i.e. using efficient building blocks through depth wise separable convolution, there are two new characteristics to the V2 architecture. The first feature is linear bottlenecks between the layers, and the second is shortcut connections between the bottlenecks. Since their classifier has good functionality on benchmarks like ILSVRC, we have included it as a survival rate classifier for this study.

##### DeepSurvNet classifier model (Fig. 1h)

After the utilization of five classifiers introduced in the previous part on the different patch sizes, the best classifier model of survival rate is selected. It should be noted since we have five classifiers and three different sizes of patches, and the number of models applied was 15 in total. The best classifier with the highest accuracy and the lowest loss among all the 15 classifiers is called DeepSurvNet.

### Evaluation criteria

Several metrics like confusion matrix [49]; the combination of precision, recall and F-score [50]; and the area under the ROC curve (AUC) [51] were used for performance evaluation of our classifiers.

#### Confusion matrix

The confusion matrix summarizes a classifier success in the prediction of examples belonging to different classes based on true positives (TP), true negatives (TN), false negatives (FN) and false positive (FP) values. This table is used to calculate the other performance metrics, i.e. precision, recall and Matthews correlation coefficient (MCC).

#### Precision, recall and F-score

Precision and recall are defined as follows:

2 $$ \Pr ecision=\frac{TP}{TP+ FP} $$

3 $$ \operatorname{Re} call=\frac{TP}{TP+ FN} $$

And F-score is the harmonic average of the precision and recall:

4 $$ F- Score=\frac{2\left(\Pr ecision\times \operatorname{Re} call\right)}{\left(\Pr ecision+\operatorname{Re} call\right)} $$

The MCC value is a correlation coefficient between the targets and predicted classifications:

5 $$ MCC=\left( TP\times TN\right)-\left( FP\times FN\right)/\sqrt{\left( TP+ FP\right)\left( TP+ FN\right)\left( TN+ FP\right)\left( TN+ FN\right)} $$

Precision, recall and F-score reach their best values at 1 and worst at 0. MCC of + 1 indicates a perfect prediction and − 1 represents completely disagreement between target and prediction.

#### Area under the curve (AUC) and receiver operating characteristics (ROC)

ROC curves combine the true positive rate (TPR or sensitivity) and false positive rate (FPR or 1-specificity) to illustrate the classification performance. These two metrics are defined as follows:

6 $$ TPR=\frac{TP}{TP+ FN} $$

7 $$ FPR=\frac{FP}{FP+ TN} $$

A perfect classifier would achieve higher AUC, and AUC of 1 means the best classification.

## Implementation details

In this study, in the preprocessing stage, for WSIs visualization and removing outliers, we have used Aperio ImageScope software. Also, we have initialized our input shapes to 224 × 224 × 3 channels (224 × 224 × 3) for all of the classifiers. After several experiences, we found that the best practices for setting parameters and hyperparameters in training stage are 30 epochs with stochastic gradient descent (SGD) optimizer, an initial learning rate of 0.01, the momentum of 0.9, learning rate decay of 0.001 and categorical cross-entropy as loss function. In order to tackle the overfitting problem, we have applied the dropout regularization technique. All the networks were implemented in python with the Keras [52], a high-level neural networks API running on Tensorflow framework [53], and trained using four NVIDIA 1080Ti GPUs.

## Results and discussion

### Survival rate classifiers comparison

**Fig. 3:**
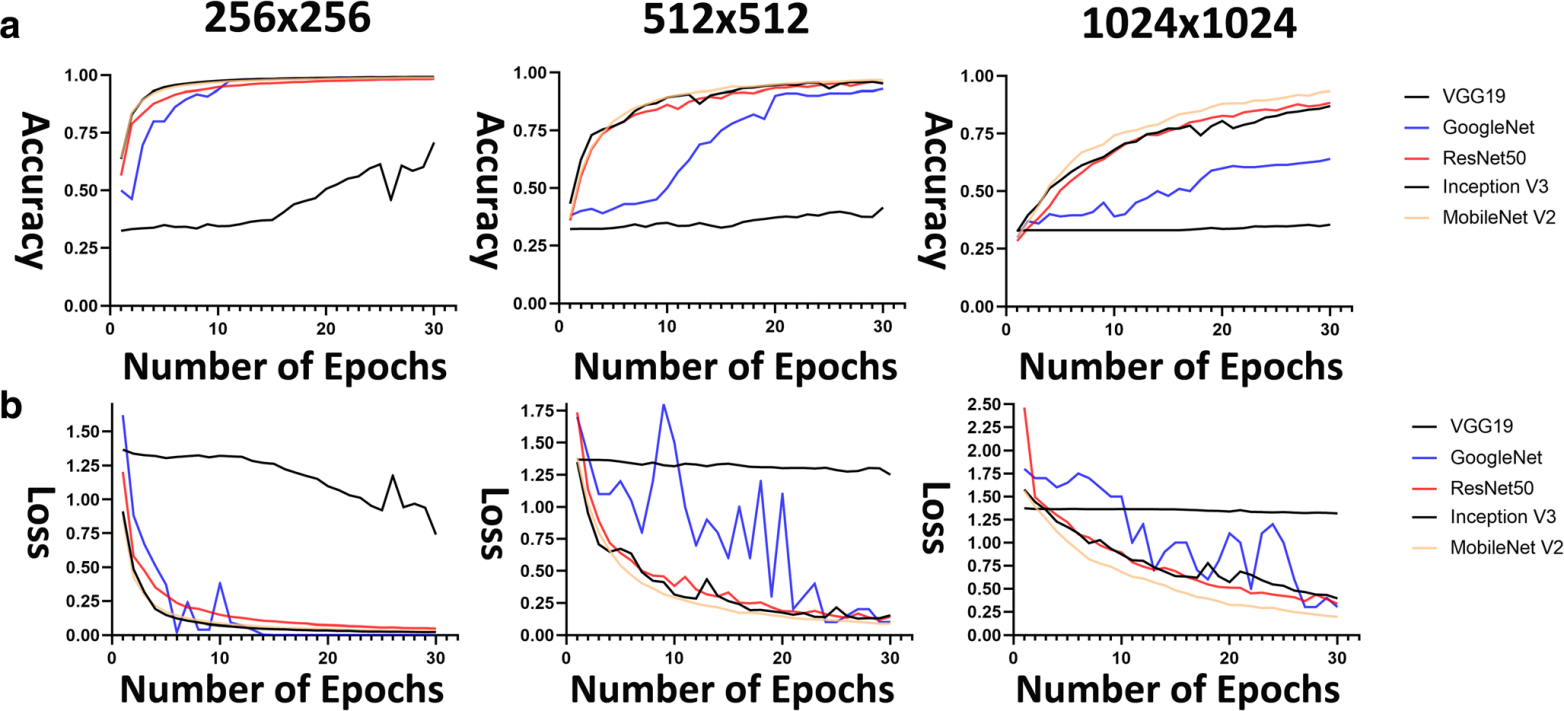
**a** Accuracy curves and **b** loss curves for five classifiers in training phase. *Left*, patches 256 × 256; *middle,* patches 512 × 512; and *right*, patches 1024 × 1024

Figure 3 shows training accuracy and loss curves in training phase for different patch sizes (256 × 256, 512 × 512 and 1024 × 1024) for all survival classifiers (note that all classifiers were applied to the same TCGA training patches). Results using 256 × 256 patch size show that for all classifiers, this size has improved training accuracy curves (nearly 1) and the lowest training loss curves (nearly 0) when compared to the other patch sizes.

Then, in the testing phase, we evaluate the “trained classifiers to the corresponding test set (i.e. a set of patch images of different sizes)”. During this phase, we calculated confusion matrix, AUC, and the achieved values for all the evaluator metrics including recall, precision and F-score for the different classifiers (Table 2). We found that using GoogleNet led to the highest level of ordered pair of (i) average precision and (ii) average AUC of 0.65 and 0.86, 0.93 and0.99 and 0.99 and1 for 1024 × 1024, 512 × 512 and 256 × 256 patch sizes, respectively. Therefore, *DeepSurvNet* classifier is powered by trained *GoogleNet* on *256 × 256* histopathological patches, given the highest average precision obtained under these conditions.

**Table 2:** Comparison between different kinds of DCNN classifiers in the testing phase

CNN model	Patch size	Class no.	Recall	F-score	Precision	MCC	Avg. Precision	Avg. MCC	Avg. AUC
VGG19	256 × 256	C1	0.61	0.61	0.61	0.57	0.65	0.62	0.87
		C2	0.57	0.59	0.61	0.64			
		C3	0.64	0.68	0.72	0.61			
		C4	0.78	0.72	0.66	0.65			
	512 × 512	C1	0.12	0.18	0.39	− 0.32	0.43	− 0.16	0.54
		C2	0.04	0.07	0.75	− 0.21			
		C3	0.83	0.38	0.25	− 0.07			
		C4	0.09	0.14	0.32	− 0.06			
	1024 × 1024	C1	0.00	0.00	0.00	− 0.52	0.42	− 0.48	0.56
		C2	0.32	0.36	0.41	− 0.52			
		C3	0.86	0.42	0.28	− 0.43			
		C4	0.16	0.28	1.00	− 0.43			
MobileNetV2	256 × 256	C1	0.70	0.76	0.84	0.51	0.81	0.54	0.95
		C2	0.82	0.80	0.78	0.54			
		C3	0.81	0.91	0.72	0.54			
		C4	0.91	0.91	0.91	0.56			
	512 × 512	C1	0.58	0.54	0.51	0.1	0.59	0.1	0.82
		C2	0.60	0.55	0.51	0.1			
		C3	0.51	0.57	0.65	0.08			
		C4	0.63	0.66	0.70	0.11			
	1024 × 1024	C1	0.00	0.00	0.00	− 0.52	0.31	− 0.43	0.53
		C2	0.66	0.40	0.28	− 0.37			
		C3	0.50	0.40	0.33	− 0.37			
		C4	0.10	0.17	0.62	− 0.47			
ResNet50	256 × 256	C1	0.82	0.85	0.87	0.63	0.85	0.64	0.96
		C2	0.81	0.86	0.90	0.63			
		C3	0.82	0.80	0.77	0.63			
		C4	0.96	0.91	0.87	0.66			
	512 × 512	C1	0.65	0.64	0.63	0.30	0.71	0.31	0.90
		C2	0.72	0.68	0.65	0.32			
		C3	0.67	0.68	0.69	0.31			
		C4	0.76	0.81	0.86	0.33			
	1024 × 1024	C1	0.24	0.33	0.55	− 0.07	0.60	0.04	0.81
		C2	0.82	0.57	0.44	0.12			
		C3	0.50	0.54	0.60	0.04			
		C4	0.72	0.77	0.84	0.1			
InceptionV3	256 × 256	C1	0.86	0.83	0.82	0.66	0.875	0.66	0.97
		C2	0.86	0.87	0.90	0.66			
		C3	0.79	0.81	0.85	0.65			
		C4	0.95	0.93	0.93	0.66			
	512 × 512	C1	0.84	0.78	0.73	0.59	0.83	0.58	0.95
		C2	0.73	0.75	0.78	0.56			
		C3	0.77	0.83	0.89	0.57			
		C4	0.96	0.94	0.92	0.61			
	1024 × 1024	C1	0.48	0.54	0.62	− 0.52	0.63	− 0.43	0.82
		C2	0.50	0.62	0.83	− 0.37			
		C3	0.76	0.58	0.47	− 0.37			
		C4	0.60	0.59	0.59	− 0.47			
GoogLeNet	256 × 256	C1	0.99	0.99	0.98	0.97	0.99	0.97	1
		C2	0.98	0.98	0.99	0.97			
		C3	0.99	0.99	0.99	0.97			
		C4	0.99	0.99	0.99	0.96			
	512 × 512	C1	0.93	0.92	0.92	0.79	0.93	0.80	0.99
		C2	0.88	0.92	0.95	0.80			
		C3	0.94	0.93	0.92	0.83			
		C4	0.96	0.94	0.93	0.78			
	1024 × 1024	C1	0.45	0.55	0.69	0.08	0.65	0.13	0.86
		C2	0.57	0.62	0.67	0.13			
		C3	0.78	0.63	0.52	0.23			
		C4	0.56	0.67	0.82	0.08			

**Fig. 4:**
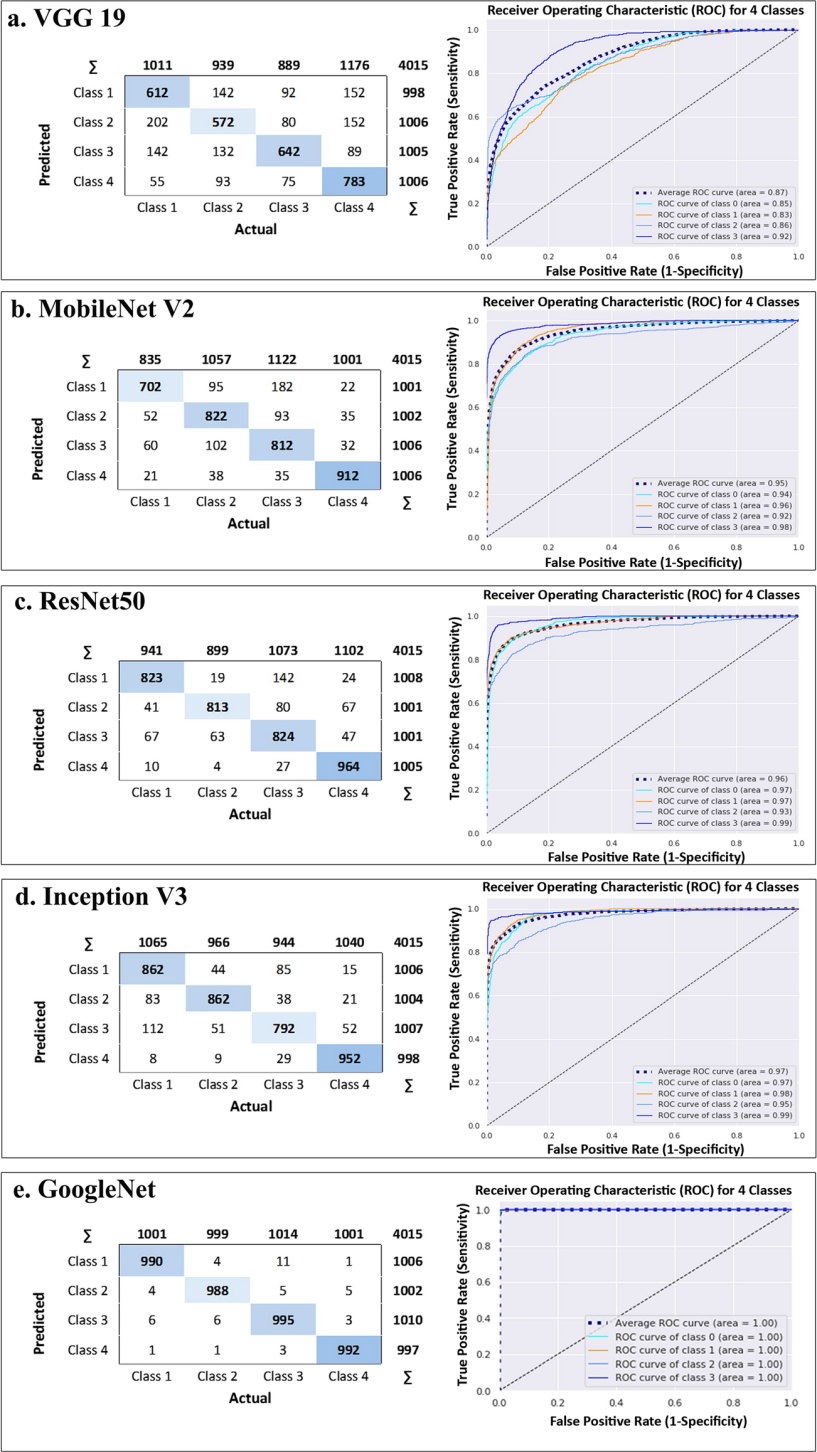
Five classifiers output on patches 256 × 256, confusion matrix in the left side and the area under ROC in right side for **a** VGG19, **b** MobileNet V2, **c** ResNet50, **d**) Inception V3 and **e** GoogleNet

Figure 4 shows the application of the 5 classifiers on 256 × 256 patch size. In this figure, confusion matrix and AUC have been depicted confirming that GoogleNet has the highest true positives and average AUC for four classes in comparison with the other classifiers. Indeed, classification results of 5 classifiers trained on 256 × 256 patch size for each cross-validation in 3 different testing folds have been shown in Table 3. The results show that the highest average indexes (among all 4 classes) including precision, recall, f1-score and MCC for all the 3 folds again are related to GoogLeNet.

**Table 3:** Classification results of 5 DCNN classifiers trained on 256 × 256 patch size for each cross validation in 3 different testing folds

	Classification results for 4 classes				
	Index (avg. on 4 classes)	**Fold #1**	**Fold #2**	**Fold #3**	Average
VGG19	Recall	0.61	0.83	0.51	0.65
	Precision	0.61	0.84	0.51	0.66
	F1-Score	0.61	0.83	0.50	0.65
	MCC	0.17	0.56	− 0.05	0.23
MobileNetV2	Recall	0.86	0.85	0.87	0.86
	Precision	0.85	0.85	0.87	0.86
	F1-Score	0.85	0.85	0.87	0.86
	MCC	0.67	0.66	0.68	0.67
ResNet50	Recall	0.84	0.86	0.60	0.77
	Precision	0.85	0.86	0.64	0.78
	F1-Score	0.84	0.86	0.60	0.58
	MCC	0.65	0.68	0.10	0.36
InceptionV3	Recall	0.87	0.87	0.87	0.87
	Precision	0.86	0.87	0.87	0.87
	F1-Score	0.87	0.87	0.87	0.87
	MCC	0.69	0.69	0.70	0.70
GoogLeNet	Recall	0.99	0.98	0.98	0.98
	Precision	0.98	0.98	0.99	0.98
	F1-Score	0.98	0.99	0.99	0.98
	MCC	0.97	0.96	0.97	0.97

### DeepSurvNet generalization in unseen (locally derived) dataset

**Fig. 5:**
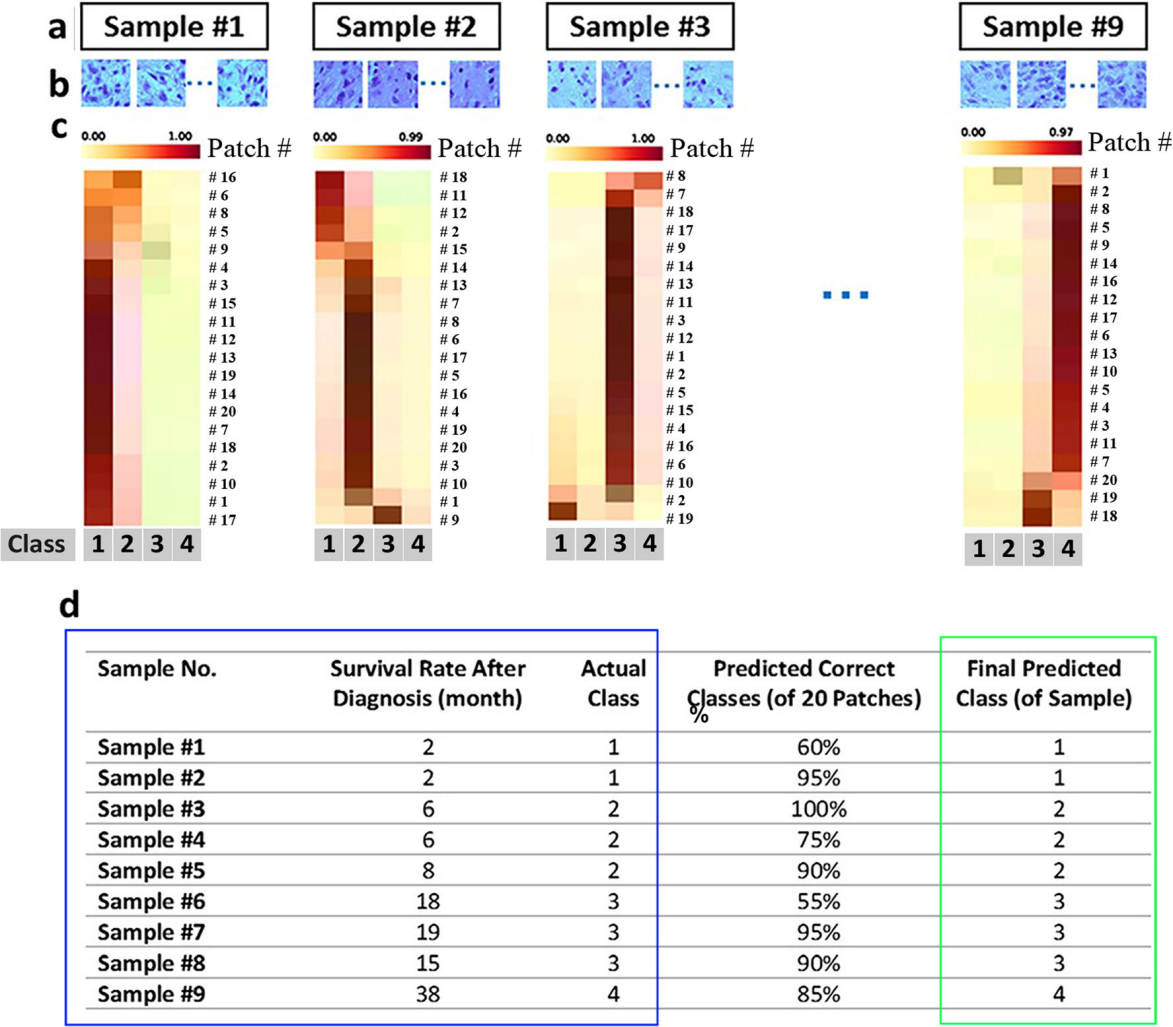
DeepSurvNet test on 9 glioblastoma patient samples (**a**) for which 20 patches were extracted from each sample (**b**). Patch classification for each patch in each sample using DeepServNet (**c**). **d** Summary of results and comparison of actual class (blue square) in patients with the corresponding predictions (3rd column, % of predicted correct classes based on the analysis of 20 patches; 4th column, class with the highest number of predicted patches, green square)

Having established a pipeline for accurate prediction for the different classes to which patient’s survival allocate based on pathological images using DeepSurvNet, we then wanted to test the accuracy of the model using a completely unseen data, which is of relevance for those who might also want to apply this pipeline with already available brain cancer histopathological slides. For this, we analysed images of H&E-stained glioblastoma tissue sections collected by SA Pathology from 9 patients undergoing tumour resection in local hospitals. Figure 5 shows the summary of the results. First, H&E histopathological images from each patient (Fig. 5a, b) were analysed in consultation with the clinical pathologist for the distinction of those regions that correspond to the tumour. These ROIs were used to extract 20 patches per patient for “patch classification” using the TCGA-trained DeepSurvNet classifier (Fig. 5b). From the different patients, we observe that the frequency of class prediction per patient was highly biased towards a single class as would be expected since patches were derived from the same pathological sample (Fig. 5c, d). Remarkably, this single class perfectly matches the real class to which patients belong (9 of 9 patients, Fig. 5d).

**Fig. 6:**
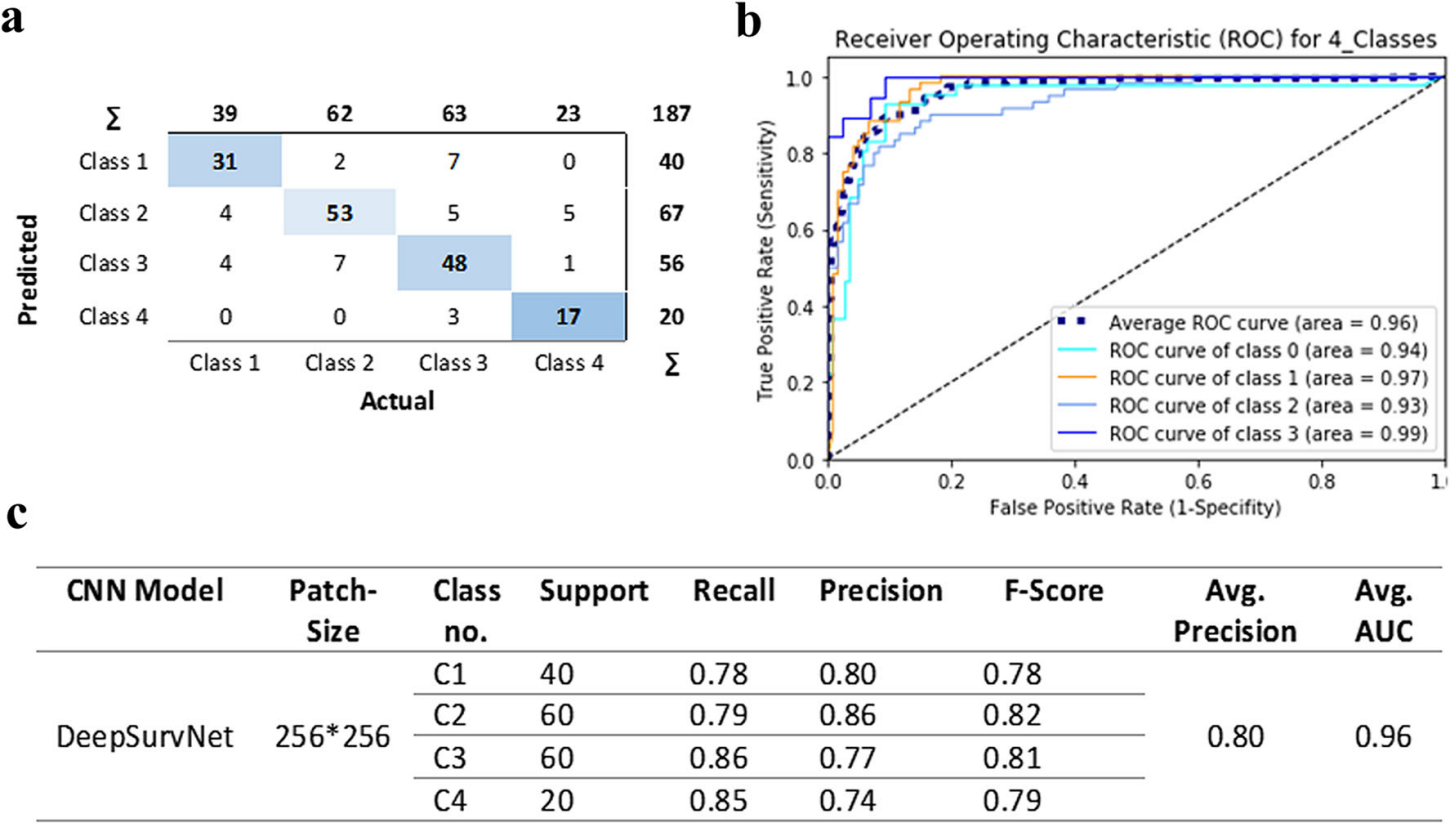
**a** Confusion matrix for total patches, **b** the area under ROC curve for all patches in four classes and **c** DeepSurvNet outputs summary for all patches

We then performed precision analysis based on (i) the analysis of 20 × 9 = 180 patches derived from these samples (i.e. without making a distinction to which patient they belong. Confusion matrix results (Fig. 6) show that the application of DeepSurvNet to this unseen dataset led to an average global precision of 80%. This precision was higher for patches belonging to class I and class II (80% and 86%, respectively) and lower for those patches belonging to class III and class IV (77% and 74%, for which morphological and genetic features are much more heterogeneous, see below).

### Gene mutation frequency within survival classes

**Fig. 7:**
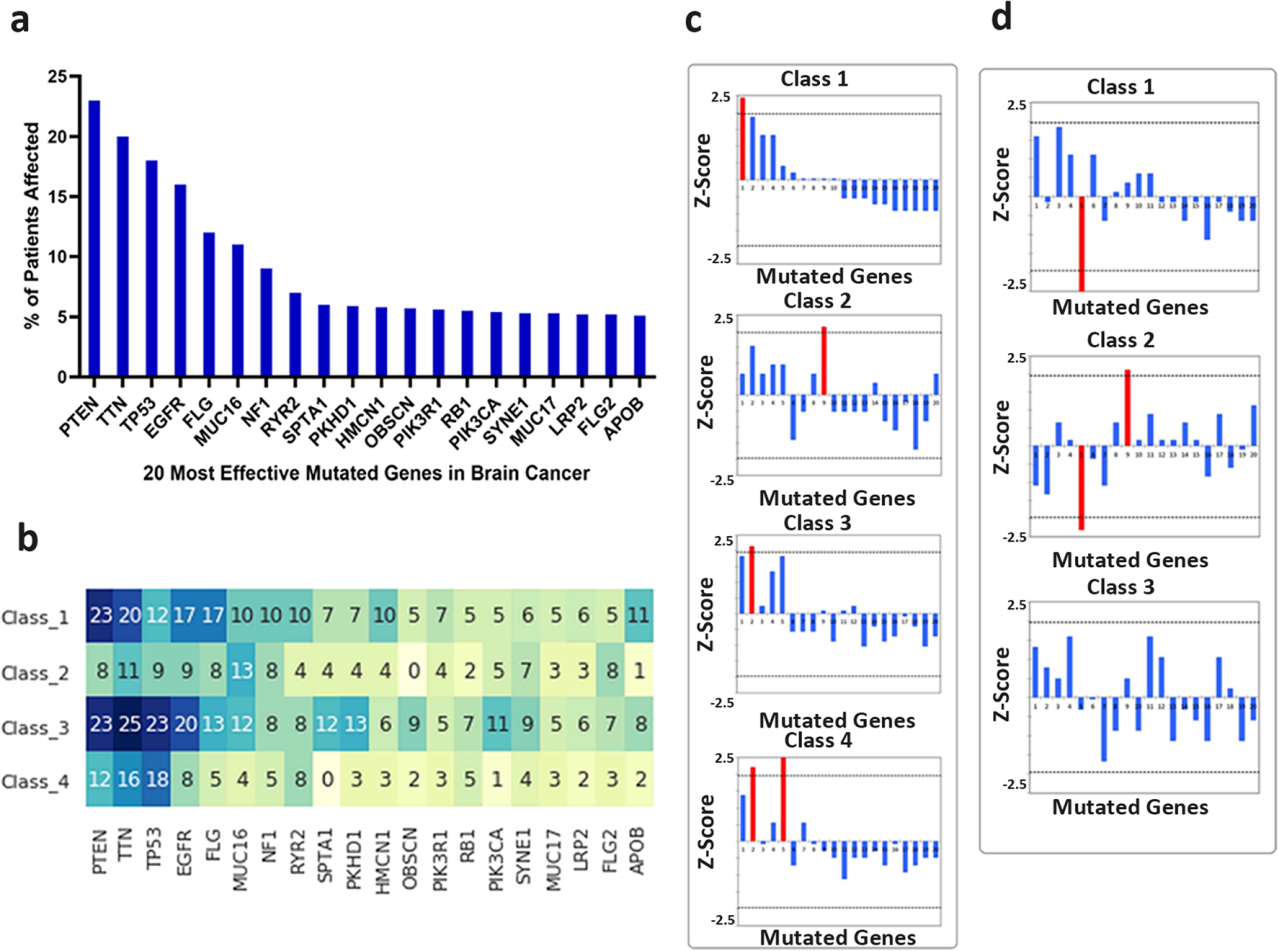
Brain cancer–mutated genes expression analysis in the four survival classes. **a** 20 most effective mutated genes in brain cancer, **b** number of patients related to each mutated genes in each class, **c** recognition of the most important gene in each class based on Z-score analysis and **d** differences in frequency of mutations of each class with respect the frequency of mutations in class IV

We then sought for better understanding of the underlying genetic differences associated with each class. For this, we analysed the distribution of frequency for mutated genes in the different survival classes using data derived from the TCGA database (Fig. 7). First, we found that by pooling all brain cancer data, the most highly mutated genes were PTEN, TTN, TP53EGFR, PLG and MUC 16 (Fig. 7a). We then analysed the frequency of mutations within each class and compared it to the distributions for all patients (Fig. 7b). We found that the distribution of gene mutations in class I mimics better than the one from the whole cohort, this being less obvious for the rest of the classes. This potentially highlights the underlying genetic differences between the classes and their impact on patient survival. To gain further insight into this, we performed a Z-score analysis to test whether there are highly mutated genes associated to each class by identifying those genes whose frequency of mutations is higher than 2 standard deviations of the frequency values for the entire set of genes (Fig. 7c). Interestingly, we found specific genes associated with each class (class I, PTEN; class II, SPTA1; class III, TTN; and class IV, TTN and FLG). Of these, the clinical significance of TTN mutations is limited since high rates of TTN mutations (passenger mutations) are mostly due to large size of this protein and variation of mutation rates across the genome [54]. We were also interested in those mutations that were different between classes, in particular, those features that are different between those patients with short and long survival. For this, we calculated the differences in frequency of mutations of each class with respect to the frequency of mutations in class IV, to discover which genes are more often aberrant in those short survival cancers (compared to those with long survival) (Fig. 7d). In particular, lack of mutations of FLG are associated with class I and class II; this adds to the presence of PTEN and SPTA mutations within these classes to define their signatures. Also, we found that there are no clear differences between long survival classes (III and IV), which highlight short survival cancers, like glioblastoma (Supplementary Table 1), are intrinsically different from those long survival cancers and correlate with our precision analysis in SA Pathology samples on which accuracy is reduced for these classes.

From the above analysis of frequency of mutated genes in brain cancer, it is worth to highlight the identification of *flg* mutations in class III and IV patients. The National Cancer Institute (NCI) is currently developing a new genomics database, the *Exceptional Responders Initiative (ERI)*, to identify molecular features of patients who have a unique response to treatments and therefore exhibit long survival rates (i.e. “exceptional responders”). FLG is a high-affinity receptor of basic fibroblast growth factor (bFGF), and a recent report by Wipfler et al. has shown that FLG has a significantly different distribution of patients affected by somatic nonsynonymous mutations. Of these, 25% of exceptional responders had one mutation each in FLG [13]. In contrast, overexpression of FLG is associated with low immune cell infiltration and short survival rates in melanoma and ovarian cancer [55], while the loss of function mutations in FLG is associated with lower cancer risk in several cancers [56]. This suggests that FLG mutations in patients with long survival rates confer a prognostic benefit possibly related to immune cell infiltration within the glioma tumour cellular microenvironment, a feature that can be detected in H&E-stained tissue sections by our image-based classifier. Similarly, SPTA1 (Spectrin, alpha, erythrocytic 1) mutations can led to alterations in H&E-stained tissue features due to its involvement in the regulation of cortical actin organization and cell shape as it has been shown in other cancers [57], although its role in GBM has not been investigated yet. Similar conclusions in relation to the tumour microenvironment and the differential expression of extracellular matrix (ECM) proteins (and therefore outside-in cell-ECM signalling) have been identified to be highly and inversely correlated to patient’s survival rates [58]. Thus, these observations suggest that differences in the cellular and noncellular microenvironment [10] and the way that cancer cells sense it through adhesion receptors and modulation of the actin cytoskeleton (i.e. EMT [59] and invasion [60]) are reflected as key biological features that could be captured by our image-based survival rate classifier.

## Conclusion

We tested the possibility of using H&E-stained brain cancer histopathological images as input data for patients’ survival classification using DCNN. In doing so, we compared the performance of DCNN algorithms using two independent datasets: the first publicly available in TCGA and the other generated by ourselves from samples collected in Adelaide. DeepSurvNet is GoogleNet classifier trained on 200,000 training samples using TCGA brain cancer dataset. Patches classification accuracy using DeepSurvNet was of 99% in the testing phase. Moreover, we found that our model DeepSurvNet classified > 50% patients’ patches class with > 90% accuracy and more than > 75% patients’ patches with 75% accuracy and 100% accuracy when considered the single patient classification based on the total patches per patient. Moreover, since for each patient the model could classify > 50% of patches in a correct class, we can also say that the classifier accuracy for 9 patients is 100%.

The analysis of frequency of mutations within these survival classes shows differences between these in terms of frequency and type of genes associated to patients with different survival rates, supporting the idea of a different genetic fingerprint associated to patient survival. This highlight that differences between short and long survival tumours and the underlying genetic characterisitcs could be useful not only in scheduling of treatments but also for the identification of new targets for glioblastoma. Thus, we conclude that DeepSurvNet constitute a new AI tool to assess the malignancy of brain cancer, which could help in the evaluation of patient treatment.

## Electronic supplementary material

Supplementary Table 1 TCGA patient ID, brain tumour type and survival. (XLSX 25 kb)
